# Linc-RAM is a metabolic regulator maintaining whole-body energy homeostasis in mice: Linc-RAM is a metabolic regulator

**DOI:** 10.3724/abbs.2022170

**Published:** 2022-11-11

**Authors:** Qianying Zhang, Lili Zhai, Qian Chen, Yixia Zhao, Ruiting Wang, Hu Li, Tian Gao, Meihong Chen, Dahai Zhu, Yong Zhang

**Affiliations:** 1 State Key Laboratory of Medical Molecular Biology Institute of Basic Medical Sciences Chinese Academy of Medical Sciences and School of Basic Medicine Peking Union Medical College Beijing 100005 China; 2 The Max-Planck Center for Tissue Stem Cell Research and Regenerative Medicine Bioland Laboratory (Guangzhou Regenerative Medicine and Health Guangdong Laboratory) Guangzhou 510005 China; 3 Shandong University of Traditional Chinese Medicine Jinan 250355 China

**Keywords:** long noncoding RNA, Linc-RAM, muscle fiber type, basal metabolic rate, metabolic homeostasis

## Abstract

Long noncoding RNAs (lncRNAs) are known to have profound functions in regulating cell fate specification, cell differentiation, organogenesis, and disease, but their physiological roles in controlling cellular metabolism and whole-body metabolic homeostasis are less well understood. We previously identified a skeletal muscle-specific long intergenic noncoding RNA (linc-RNA) activator of myogenesis, Linc-RAM, which enhances muscle cell differentiation during development and regeneration. Here, we report that Linc-RAM exerts a physiological function in regulating skeletal muscle metabolism and the basal metabolic rate to maintain whole-body metabolic homeostasis. We first demonstrate that Linc-RAM is preferentially expressed in type-II enriched glycolytic myofibers, in which its level is more than 60-fold higher compared to that in differentiated myotubes. Consistently, genetic deletion of the *Linc-RAM* gene in mice increases the expression levels of genes encoding oxidative fiber versions of myosin heavy chains and decreases those of genes encoding rate-limiting enzymes for glycolytic metabolism. Physiologically, *Linc-RAM*-knockout mice exhibit a higher basal metabolic rate, elevated insulin sensitivity and reduced fat deposition compared to their wild-type littermates. Together, our findings indicate that Linc-RAM is a metabolic regulator of skeletal muscle metabolism and may represent a potential pharmaceutical target for preventing and/or treating metabolic diseases, including obesity.

## Introduction

Eukaryotic genomes are extensively transcribed to produce long noncoding RNAs (lncRNAs) in a temporally and spatially regulated manner [ 1, 2] . An increasing number of lncRNAs have been reported to have profound functions in regulating cell lineage differentiation, cell proliferation, and tumorigenesis during development and in various pathological settings [ 3‒ 5] . For example, in pluripotent cells, the divergent lncRNA Evx1as promotes the transcription of its neighboring gene, *EVX1*, to regulate mesendodermal differentiation [6]. The human-specific lincRNAs govern neuronal lineage commitment and contribute to human striatum development [7]. The lncRNA Handsdown (Hdn) regulates the cardiac gene program and is essential for early mouse development [8]. The myeloid-specific lncRNA LOUP originates from the upstream regulatory element of the *PU*. *1* gene and induces myeloid differentiation by acting as a transcriptional inducer of the myeloid master regulator PU.1 [9].

Mechanistically, lncRNAs function as fundamental transcription and posttranscription regulators; and they act at multiple levels of gene expression in *cis* and/or *trans* in the nuclear and/or cytoplasmic compartments. Some experimental data suggest that lncRNAs function via regulating cell metabolism [10]. The lncRNA breast cancer anti-estrogen resistance 4 (BCAR4) reprograms glucose metabolism by upregulating the transcription of glycolysis-related genes in cancer cells [11]. The lncRNA GLCC1 is upregulated under glucose starvation in colorectal cancer cells to support cell survival and proliferation by enhancing glycolysis [12]. The lncRNA NEAT1 critically contributes to metabolic changes during breast cancer growth and metastasis by regulating the penultimate step of glycolysis [13]. LncRNA-ACOD1, which is identified by the nearby gene encoding aconitate decarboxylase 1 ( *Acod1*), significantly attenuates viral infection by directly binding to the metabolic enzyme glutamic-oxaloacetic transaminase (GOT2) and enhancing its catalytic activity [14].

Skeletal muscle accounts for 40%‒45% of the body mass and functions as an important metabolic and endocrine organ to orchestrate the basal metabolic rate [ 15, 16] . It plays pivotal roles in regulating whole-body metabolic homeostasis by actively communicating with other metabolic organs, such as fat and liver [ 16‒ 18] . Emerging studies have documented that lncRNAs regulate skeletal muscle cell differentiation during development and regeneration [19]. For example, the lncRNA Linc-MD1 has been shown to control muscle cell differentiation in both mouse and human myoblasts [ 20, 21] . Although great progress has been made in elucidating the functions of lncRNAs in regulating muscle cell differentiation, we know relatively little about whether and how lncRNAs control muscle metabolism. Recent studies have shown that the lncRNA H19 acts to enhance muscle insulin sensitivity by activating AMPK [22]. The administration of H19 RNA gain-of-function oligonucleotides (H19-Rgof) was found to improve muscle mass, muscle performance, and the basal metabolic rate in mice. Furthermore, mice treated with H19 RNA reportedly resisted HFD- or leptin deficiency-induced obesity [23].

We previously identified and characterized a long intergenic noncoding RNA (linc-RNA) activator of myogenesis (Linc-RAM), which is specifically expressed in skeletal muscle cells and localized in both the nucleus and the cytoplasm [ 24, 25] . Nuclear Linc-RAM promotes muscle cell differentiation by facilitating the assembly of the MyoD-Baf60c-Brg1 complex on the regulatory elements of target genes [24]. Cytoplasmic Linc-RAM contributes to muscle cell differentiation by directly interacting with glycogen phosphorylase (PYGM) and modifying PYGM activity during myogenic differentiation [25]. Linc-RAM is transcriptionally regulated by MyoD via the FGF2/Ras/Raf/MEK/Erk signaling pathway during muscle cell differentiation [26]. However, the expression and physiological function(s) of Linc-RAM in fully differentiated mature skeletal muscle remain to be revealed.

In the present study, we report a physiological function of Linc-RAM in regulating skeletal muscle metabolism and the basal metabolic rate to maintain whole-body metabolic homeostasis. Our findings suggest that Linc-RAM may represent a potential pharmaceutical target for preventing and/or treating metabolic diseases, including obesity.

## Materials and Methods

### Mouse lines and animal care

All animal experiment procedures were approved by the Animal Ethics Committee of Peking Union Medical College (Beijing, China). Mice were housed in a pathogen-free facility and had free access to water and standard rodent chow under the following conditions: 21°C ambient temperature, 50%–60% humidity, and 12/12-h dark/light cycle. The *Linc-RAM*-knockout mice in the C57BL/6j background were produced as previously described [24]. Two-month-old and 18-month-old male *Linc-RAM-*knockout and wild-type littermate mice were used in the study.

### Primary myoblast isolation, culture, and differentiation

Primary myoblasts were isolated from hind-limb skeletal muscles of C57BL/6j mice at 2–3 weeks old, minced, and digested in a mixture of type II collagenase and dispase. Cells were filtered from debris and centrifuged, and fibroblasts were eliminated by differential attachment for 2× 10 min. The obtained cells were cultured in F-10 Ham’s medium (Cat. No. CM10070; M&C Gene Technology, Beijing, China) supplemented with 20% fetal bovine serum (Cat. No. vs500T; Ausbian, Sydney, Australia), 10 ng/mL basic fibroblast growth factor (Cat. No. 10014-HNAE; Sino Biological, Beijing, China), and 1% antibiotics (Cat. No. 13-0050; ZellShleld, Berlin, Germany) on collagen-coated cell culture plates at 37°C in 5% CO _2_. For the differentiation of primary myoblasts, cells were transferred to Dulbecco’s modified Eagle’s medium (Cat. No. C11995500BT; Gibco, Carlsbad, USA) containing 2% horse serum (Cat. No. SH30074.03; HyClone, Logan, USA) and 1% penicillin (Cat. No. 0242; Amresco, Washington, USA)and streptomycin (Cat. No. 0382; Amresco, Washington, USA ) and then cultured for the indicated time (1 day, 1 d; and 2 days, 2 d). All cells were grown to 80% confluence before induction of differentiation.

### Real-time quantitative reverse transcription-polymerase chain reaction (RT-qPCR)

Trizol reagent (Invitrogen, Carlsbad, USA) was used to extract total RNA from proliferating myoblasts, differentiated myotubes, or various skeletal muscles, including *gastrocnemius* (Gas), *quadriceps* *femoris* (Qu), *extensor digitorum longus* (EDL), *tibialis anterior* (TA), and *soleus* (Sol). Total RNA was reverse-transcribed with reverse transcriptase (TaKaRa, Dalian, China). Real-time quantitative PCR analyses were performed in triplicate using Fast Eva Green qPCR Master Mix (Bio-Rad, Hercules, USA). *β-Actin* was used as an internal control for RT-qPCR analyses. All primers used for RT-qPCR are presented in Table 1.

**Table:** **Table 1** Sequences of primers used in the study

Gene	Primer sequence
*β-actin*	F: 5′-CTGGCTGGCCGGGACCTGAC-3′
	R: 5′-CCGCTCGTTGCCAATAGTGATGAC-3′
*MyoG*	F: 5′-CCATTCACATAAGGCTAACAC-3′
	R: 5′-CCCTTCCCTGCCTGTTCC-3′
*MyHC*	F: 5′-CTTGGTGGACAAACTACAGACT-3′
	R: 5′-TGCAGAATTTATTTCCGTGAT-3′
*Myh7*	F: 5′-CCTTGGCACCAATGTCCCGGCTC-3′
	R: 5′-GAAGCGCAATGCAGAGTCGGTG-3′
*Myh1*	F: 5′-AAGGAGCAGGACACCAGCGCCCA-3′
	R: 5′-ATCTCTTTGGTCACTTTCCTGCT-3′
*Myh2*	F: 5′-ATGAGCTCCGACGCCGAG-3′
	R: 5′-TCTGTTAGCATGAACTGGTAGGCG-3′
*Myh4*	F: 5′-GTGATTTCTCCTGTCACCTCTC-3′
	R: 5′-GGAGGACCGCAAGAACGTGCTGA-3′
*Linc-RAM*	F: 5′-GAACCAACGTTGCTAGGAGA-3′
	R: 5′-CTGAGAGCCTCAGGAGGTAG-3′
*HK2*	F: 5′-AACCTCAAAGTGACGGTGGG-3′
	R: 5′-TCACATTTCGGAGCCAGATCT-3′
*PFKm*	F: 5′-CAGATCAGTGCCAACATAACCAA-3′
	R: 5′-CGGGATGCAGAGCTCATCA-3′
*PKm*	F: 5′-CGATCTGTGGAGATGCTGAA-3′
	R: 5′-AATGGGATCAGATGCAAAGC-3′

### Immunofluorescence staining

For cryosections of *soleus* muscle, the slides were incubated in 1.0% Triton X-100 in PBS at room temperature for 10 min. Subsequently, the sections were incubated at room temperature for 1 h in filtered blocking buffer (4% BSA, and 0.1% Triton X-100). The primary antibodies were diluted with PBS buffer containing 4% BSA. Monoclonal anti-myosin (skeletal, Fast) was purchased from Sigma (M1570; 1:200; St Louis, USA). Primary antibodies were loaded onto a specimen and incubated overnight at 4°C. Then, the slides were washed with PBS containing 0.1% BSA and incubated for 1 h with fluorescein-conjugated secondary antibodies (1:200; Zhongshanjinqiao Corporation, Beijing, China). After wash several times with PBS, the samples were imaged under a fluorescence microscope (Olympus, Tokyo, Japan).

### Metabolic chamber analysis

Metabolic phenotyping of standard diet-fed wild-type and *Linc-RAM*-knockout mice was performed with the Oxymax/CLAMS metabolic cage system (Columbus Instruments, Columbus, USA) at the Animal Center of Peking Union Medical College. The mice had free access to water and standard rodent chow under a 12/12 h dark/light cycle. Food intake, drinking, O _2_ consumption, and CO _2_ production were automatically collected for 4 consecutive days.

### Glucose- and insulin-tolerance tests

Overnight-fasted mice were given intraperitoneal (i.p.) injections of glucose (2 mg/g body weight) for the glucose tolerance test (GTT). For the insulin tolerance test (ITT), mice were fasted for 4 h and then given 1 mU insulin/g body weight (Novolin, Tianjin, China) by i.p. injection. Blood glucose was determined with a Lifescan One Touch glucometer (Cat. No. G7021-1KG; Sigma).

### Statistical analysis

Data are presented as the mean±SEM. For statistical comparisons of two conditions, the two-tailed Student’s *t* test was used. Statistical analyses were performed using GraphPad Prism software. *P*<0.05 was considered statistically significant.

## Results

### Linc-RAM is predominantly expressed in type II-enriched muscle groups of mice

**Figure 1:**
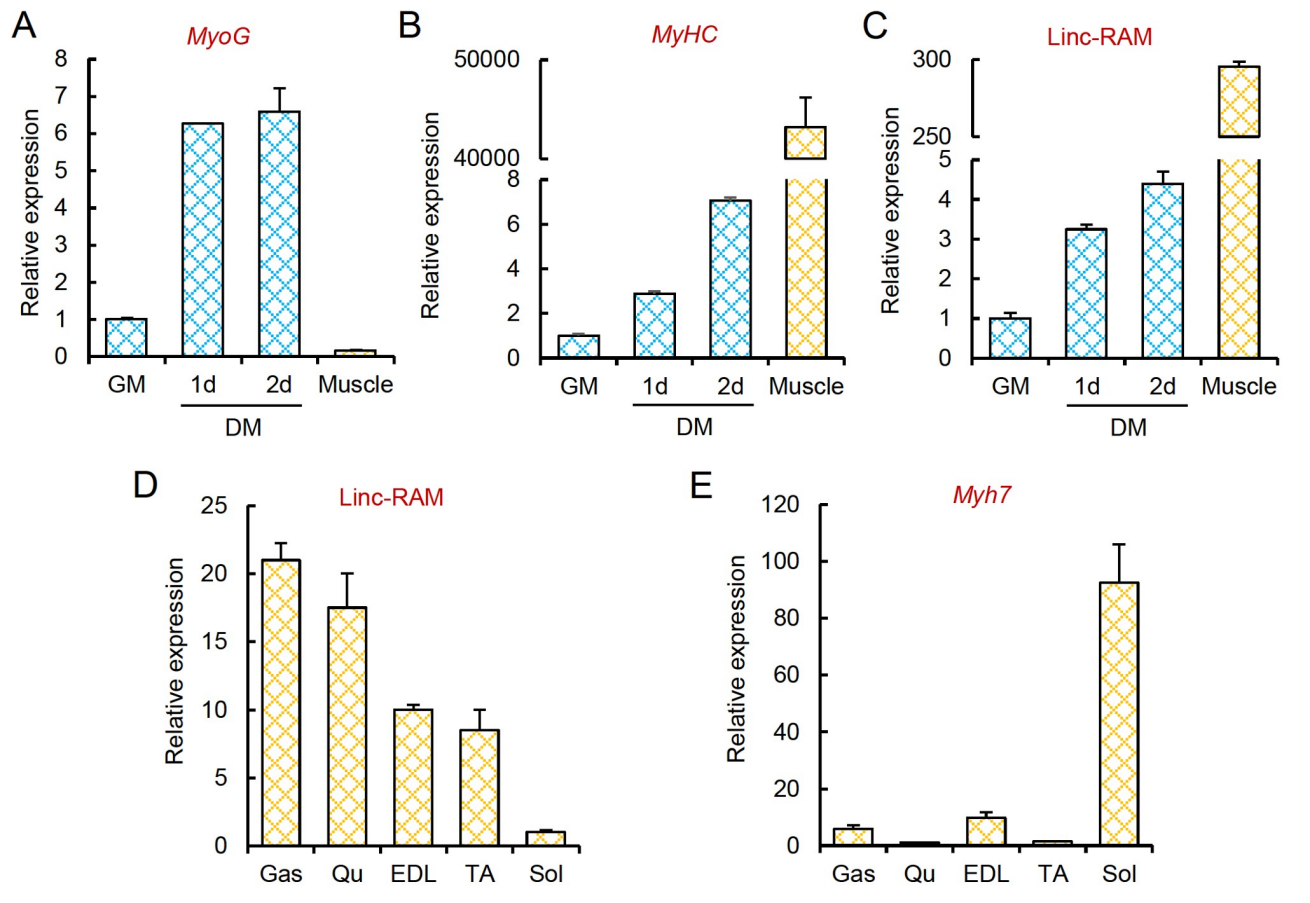
Figure 1 Linc-RAM is predominantly expressed in type II-enriched muscle groups in mice (A‒C) Relative expressions of MyoG (A), myosin heavy chain ( MyHC) (B) and Linc-RAM (C) in proliferating myoblasts cultured in growth medium (GM), differentiated muscle cells cultured in differentiation medium (DM) for the indicated days (1 d and 2 d), and mature skeletal muscle ( tibialis anterior), as determined by RT-qPCR. (D) Relative expression of Linc-RAM in various muscle groups from mice, including gastrocnemius (Gas), quadriceps femoris (Qu), extensor digitorum longus (EDL), tibialis anterior (TA), and soleus (Sol), as determined by RT-qPCR. (E) Relative expression of myosin heavy chain-encoding Myh7 (encoding MyHC-I) in the indicated muscle groups from mice, as determined by RT-qPCR. β-Actin served as an internal control. Data are presented as the mean±SEM, n=4 per group.

We previously identified a skeletal muscle-specific lncRNA, Linc-RAM, which functions in regulating muscle cell differentiation [24]. To investigate the physiological function of Linc-RAM in mature skeletal muscle, we examined Linc-RAM expression in undifferentiated myoblasts, differentiated myotubes, and fully differentiated mature skeletal muscle ( *tibialis anterior*). First, *MyoG* and myosin heavy chain ( *MyHC*) expressions indicated that the cells were well differentiated ( Figure 1A,B). Linc-RAM was expressed in proliferating myoblasts cultured in growth medium (GM) and significantly upregulated when the cells were shifted to differentiation medium (DM) to undergo differentiation ( Figure 1C). These findings were consistent with our previous observations [24]. We further found that the expression level of Linc-RAM was 60-fold higher in mature skeletal muscle than in 2-day differentiated myotubes ( Figure 1C), suggesting that Linc-RAM is not functionally restricted to the regulation of muscle cell differentiation but rather may also play a role in mature skeletal muscle.

Skeletal muscle comprises two types of myofibers that are distinguished by their contraction features: type I (slow-twitch) and type II myofibers (fast-twitch) [27]. The percentages of the myofiber types differ across various muscle groups and can adaptively change under physiological or pathological conditions [27]. To understand the physiological function of Linc-RAM in mature skeletal muscle, we examined its expression in various muscle groups from mice, including *gastrocnemius* (Gas), *quadriceps* *femoris* (Qu), *extensor digitorum longus* (EDL), *tibialis anterior* (TA), and *soleus* (Sol). We found that Linc-RAM was highly expressed in Gas, Qu, EDL, and TA muscles ( Figure 1D), which are enriched for type II myofibers [28]. In contrast, its expression level was low in Sol muscle, which is enriched for type I myofibers ( Figure 1D,E). Together, these findings indicate that Linc-RAM is predominantly expressed in type II-enriched muscle groups of mice.

### Linc-RAM regulates fiber type and muscle metabolism in mice

**Figure 2:**
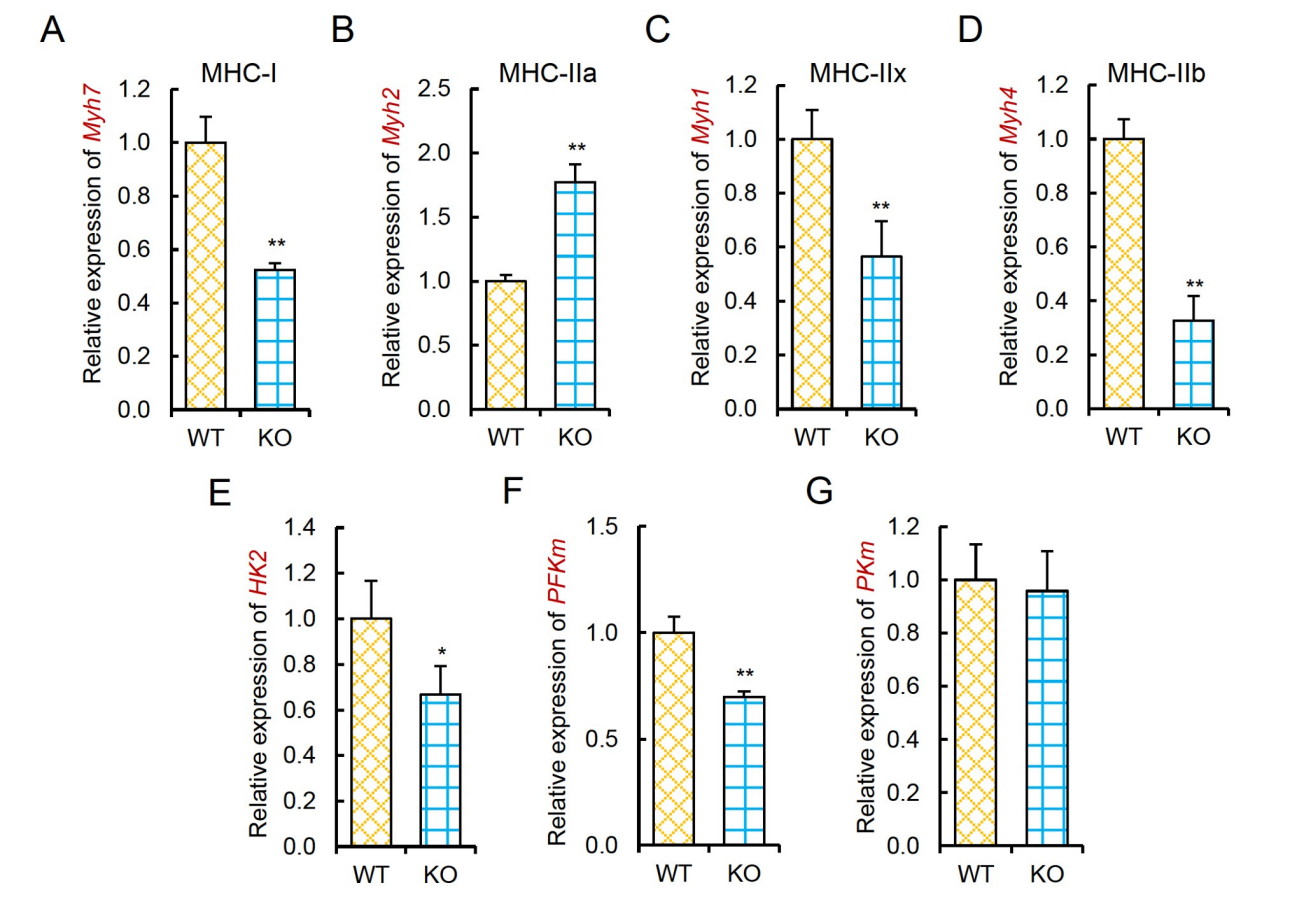
Figure 2 Linc-RAM regulates fiber type and muscle metabolism in young mice (A‒D) Relative expression levels of Myh7 (encoding MyHC-I) (A), Myh2 (encoding MHyC-IIa) (B), Myh1 (encoding MyHC-IIx) (C), and Myh4 (encoding MHyC-IIb) (D) in soleus muscle from Linc-RAM-knockout (KO) and wild-type (WT) mice at 2 months of age, as determined by RT-qPCR. (E‒G) Relative expression levels of hexokinase 2 ( HK2), muscle type phosphofructokinase ( PFKm), and muscle type pyruvate kinase ( PKm) in the same samples described in (A‒D), as determined by RT-qPCR. β-Actin served as an internal control. Data are presented as the mean±SEM, n=5 per group. * P<0.05, ** P<0.01. Two-tailed Student’s t test.

**Figure 3:**
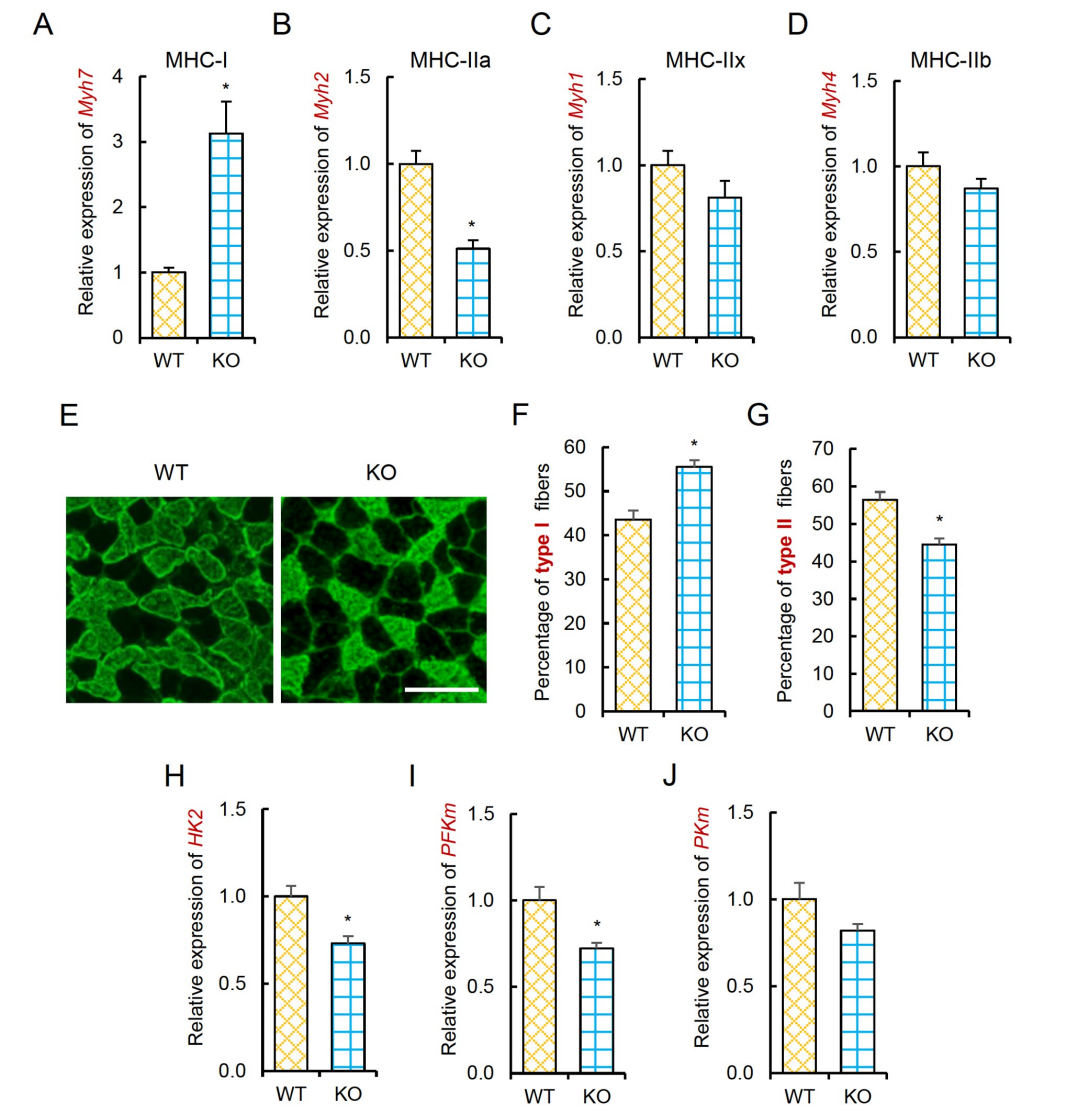
Figure 3 Linc-RAM regulates fiber type and muscle metabolism in aged mice (A‒D) Relative expression levels of Myh7 (A), Myh2 (B), Myh1 (C), and Myh4 (D) in soleus muscle from Linc-RAM-KO and WT littermates at 18 months of age, as determined by RT-qPCR. (E) Representative images of immunofluorescence staining of type II (fast-twitch) myosin heavy chain on cryosections of soleus muscle from the same mice described in (A‒D). Scale bar= 100 μm. (F‒G) Percentages of type I (F) and type II (G) myofibers, calculated on cross-sections described in (E). Five mice were examined for each genotype. (H‒J) Relative expression levels of HK2, PFKm, and PKm in the same samples described in (A‒D), as determined by RT-qPCR. β-Actin served as an internal control. Data are presented as the mean±SEM, n=5 per group. * P<0.05. Two-tailed Student’s t test.

The preferential expression of Linc-RAM in type II-enriched muscle groups suggested that Linc-RAM may function in regulating the muscle fiber type. We therefore analyzed fiber-type changes in the soleus muscle from *Linc-RAM*-knockout (KO) mice and compared to those of wild-type (WT) littermates at 2 months of age (young adult stage) ( Figure 2) and 18 months of age (aged stage) ( Figure 3). The expression levels of genes encoding various versions of fiber type-specific myosin-heavy chain (MyHC), namely, *Myh7* (encoding type I MyHC, MyHC-I), *Myh2* (encoding type IIa MyHC, MyHC-IIa), *Myh1* (encoding type IIx MyHC, MyHC-IIx), and *Myh4* (encoding type IIb MyHC, MyHC-IIb), were examined in skeletal muscle from WT and KO mice. We found that *Linc-RAM*-KO mice exhibited reduced expressions of *Myh7*, *Myh1* and *Myh4*, and elevated expression of *Myh2* in young adult mice ( Figure 2A‒D). In aged mice, *Linc-RAM*-KO mice showed reduced expressions of *Myh2*, *Myh1* and *Myh4* and elevated expression of *Myh7* ( Figure 3A‒D). This finding indicated that *Linc-RAM* knockout decreased type IIx and type IIb myofibres at both the young and aged stages, and increased type IIa myofibres at the young stage but increased type I myofibres at the aged stage. To further corroborate the fiber type alteration in *Linc-RAM*-KO mice, we performed immunofluorescence staining of fast-twitch myofibers on cryosections of *soleus* muscle from the KO and WT mice at the aged stage ( Figure 3E). We found that the percentage of type I myofibers was significantly increased in the KO mice compared to the WT controls ( Figure 3F), whereas the percentage of type II myofibers was significantly decreased in the KO mice compared to that in the WT controls ( Figure 3G).

Given that type IIx and type IIb fibers are associated with active glycolytic metabolism [27], we next measured whether *Linc-RAM* knockout decreases glycolytic activity in skeletal muscle. To this end, we assayed the expression levels of genes encoding three rate-limiting enzymes of the glycolytic pathway: hexokinase 2 ( *HK2*), muscle type phosphofructokinase ( *PFKm*), and muscle type pyruvate kinase ( *PKm*). The expression levels of *HK2* and *PFKm* were significantly decreased in *Linc-RAM*-KO mice compared to those in WT littermates at both young and aged stages ( Figure 2E‒G and Figure 3H‒J), which is consistent with the fiber-type alterations observed in *Linc-RAM*-KO mice ( Figure 2A‒D and Figure 3A‒G). Collectively, our data reveal that Linc-RAM regulates fiber type and muscle metabolism in mice.

### *Linc-RAM* knockout slightly increases O _2_ consumption and elevates insulin sensitivity at the young adult stage

**Figure 4:**
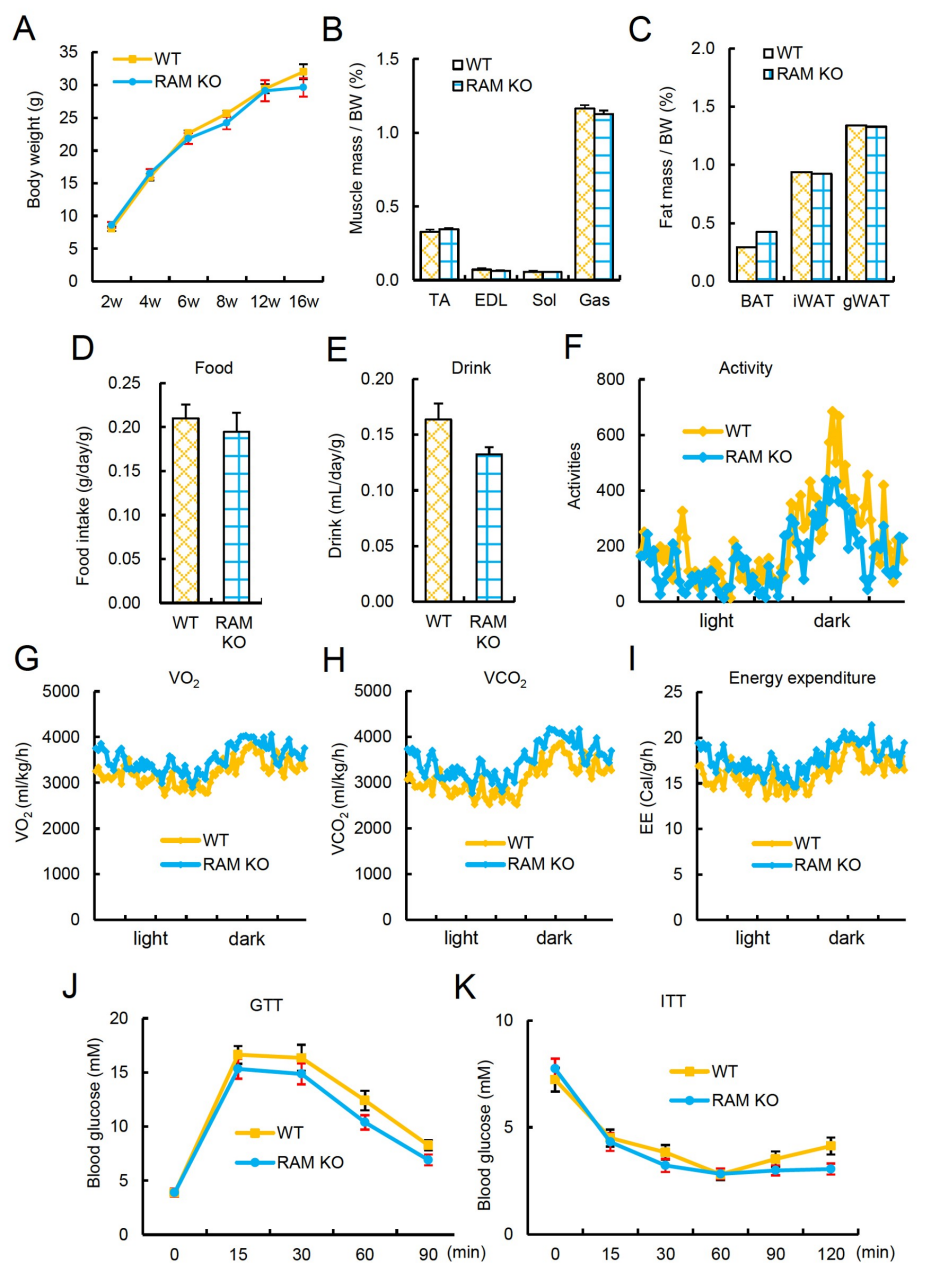
Figure 4 *Linc-RAM* knockout increases O _2_ consumption and elevates insulin sensitivity at the young adult stage Male Linc-RAM-KO and WT littermates fed with standard diet were examined at 2 months of age. (A) Growth curves for the two groups of mice, generated from weekly body weight checks (w). (B) Muscle mass normalized to body weight (BW) for the indicated muscles from the two groups of mice. (C) Fat mass normalized by BW for the indicated fat deposits of the two groups of mice. WAT, white adipose tissue (); iWAT, inguinal WAT; gWAT: gonadal WAT; and BAT, brown adipose tissue . (D) Food intake of the two groups. (E) Water intake of the two groups. (F) Activity of the two groups, as measured by metabolic chamber analysis. (G‒I) O 2 consumption (G), CO 2 production (H), and energy expenditure (EE) (I) of the two groups, as measured by metabolic chamber analysis. (J) Glucose tolerance test (GTT) results of the two groups. (K) Insulin tolerance test (ITT) results of the two groups. Data are presented as the mean±SEM, n=5 per group.

To test whether Linc-RAM-mediated muscle metabolism plays a role in maintaining whole-body metabolic homeostasis, we next performed metabolic chamber analysis on *Linc-RAM*-KO mice and WT littermates at the young adult stage. The two groups of mice did not differ overtly in body weight ( Figure 4A), muscle mass ( Figure 4B), or fat mass ( Figure 4C). No significant differences were found in the amounts of food intake ( Figure 4D), water intake ( Figure 4E) and activity level ( Figure 4F) between the two groups of mice. Interestingly, metabolic chamber analysis demonstrated that *Linc-RAM*-KO mice had slight increases in O _2_ consumption ( Figure 4G), CO _2_ production ( Figure 4H), and energy expenditure (EE) ( Figure 4I).

Given that skeletal muscle functions as an important target organ of insulin signaling and plays pivotal roles in maintaining the blood glucose level [16], we performed glucose tolerance test (GTT) and insulin tolerance test (ITT) in the *Linc-RAM*-KO mice and WT littermates. We found that *Linc-RAM*-KO mice showed slightly improved glucose tolerance ( Figure 4J) and insulin sensitivity ( Figure 4K) compared to WT littermates. Thus, our experimental data reveal that *Linc-RAM* regulates glucose metabolism in skeletal muscle in mice.

### Deletion of *Linc-RAM* increases the basal metabolic rate and reduces fat deposition in aged mice

**Figure 5:**
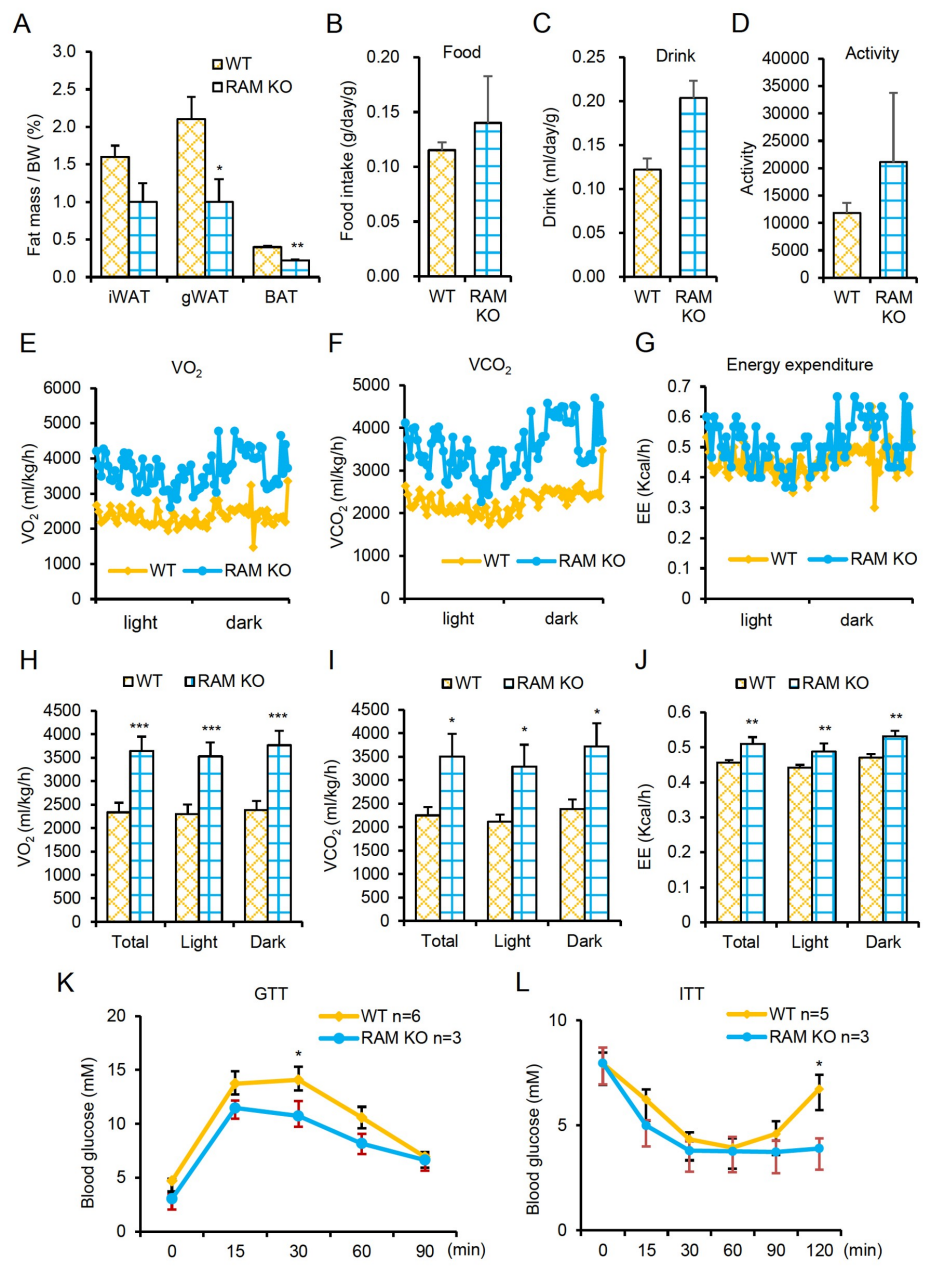
Figure 5 *Linc-RAM* knockout increases the basal metabolic rate and reduces fat deposition in aged mice Male Linc-RAM-KO and WT littermates fed with standard diet were examined at 18 months of age. (A) Fat mass normalized by BW for the indicated fat deposits from the two groups of mice. (B) Food intake of the two groups. (C) Water intake of the two groups. (D) Activity of the two groups, as measured by metabolic chamber analysis. (E‒J) O 2 consumption (E,H), CO 2 production (F,I), and energy expenditure (EE) (G,J) of the two groups, as measured by metabolic chamber analysis. (K) GTT results of the two groups. (L) ITT results of the two groups. Data are presented as the mean±SEM, n=5 per group. * P<0.05, ** P<0.01, *** P<0.001. Two-tailed Student’s t test.

Next, we examined whether *Linc-RAM* knockout influences whole-body metabolic homeostasis in 18-month-old *Linc-RAM*-KO mice and WT littermates fed with standard diet. We found that *Linc-RAM* KO mice exhibited a significantly reduced fat mass compared to WT littermates ( Figure 5A) and that this lean phenotype was not due to any between-group difference in the amount of food intake, water intake ( Figure 5B,C) or activity level ( Figure 5D).

To further explore the mechanism underlying the lean phenotype of the *Linc-RAM*-KO mice, we conducted metabolic chamber analysis on the aged mice. Our results revealed that *Linc-RAM*-KO mice exhibited significantly increased O _2_ consumption ( Figure 5E,H), CO _2_ production ( Figure 5F,I), and energy production (EE) ( Figure 5G,J) compared to their WT littermates.

As the lean phenotype is generally positively correlated with insulin sensitivity [16], we performed GTT and ITT in the aged *Linc-RAM*-KO mice and WT littermates. The GTT results showed that *Linc-RAM*-KO mice had significantly improved glucose tolerance ( Figure 5K) and significantly increased insulin sensitivity ( Figure 5L) compared to WT littermates. Together, our findings indicate that deletion of *Linc-RAM* enhances fat combustion and contributes to the lean phenotype observed in the aged *Linc-RAM*-KO mice, suggesting that *Linc-RAM* plays a regulatory role in maintaining whole-body metabolic homeostasis by controlling the basal metabolic rate in aged mice.

## Discussion

Recent studies have documented that lncRNAs act as key regulators of cell differentiation, cell lineage choice, organogenesis, and tissue homeostasis [ 3, 4] . However, the physiological roles of lncRNAs in regulating cellular metabolism and whole-body metabolic homeostasis are less well understood. Here, we report that Linc-RAM has a physiological function in regulating skeletal muscle metabolism and the basal metabolic rate to maintain whole-body metabolic homeostasis.

First, we demonstrated that Linc-RAM is preferentially expressed in type II-enriched muscle groups under physiological conditions. We previously observed that Linc-RAM is regulated by the transcription factor MyoD [24], which is expressed at a higher level in type II-enriched glycolytic TA muscle than in type I-enriched oxidative Sol muscle [29]. Accordingly, the expression level of Linc-RAM is significantly reduced in *MyoD*-knockout muscle [ 24, 26] . Consistent with these expression patterns, we herein found that, similar to MyoD, Linc-RAM functions to regulate muscle fiber type. We show that *Linc-RAM*-knockout mice exhibit elevated expressions of genes encoding the oxidative myofiber versions of myosin heavy chain, *Myh2* in young mice or *Myh7* in aged mice, suggesting that deletion of *Linc-RAM* consistently increases oxidative metabolism and decreases glycolytic metabolism in both young and aged mice. Previous work revealed that *MyoD* gene knockout also increases the percentage of oxidative myofibers [30]. Yu *et al*. [31] demonstrated that lncRNA-FKBP1C regulates muscle fiber type by directly interacting with MYH1B and enhancing its protein stability. In contrast to the functions of Linc-RAM and MyoD, knockdown of LncRNA-FKBP1C was found to drive a fiber type switch from slow-twitch muscle fibers to fast-twitch muscle fibers [31]. Thus, our data and previous reports collectively suggest that lncRNAs play important roles in regulating myofiber type and skeletal muscle metabolism.

Skeletal muscle, as an important metabolic and endocrine organ, plays pivotal roles in regulating whole-body metabolic homeostasis by actively communicating with other metabolic organs via muscle-liver or muscle-fat crosstalk [ 16‒ 18] . Here, we found that Linc-RAM-mediated muscle metabolism controls the basal metabolic rate and maintains metabolic homeostasis. We also revealed that *Linc-RAM*-knockout mice exhibit a higher basal metabolic rate, elevated insulin sensitivity, and reduced fat deposition than their wild-type littermates, highlighting that Linc-RAM-mediated muscle metabolism plays critical roles in orchestrating whole-body metabolic homeostasis. Previous work showed that the skeletal muscle-enriched lncRNA H19 enhances muscle insulin sensitivity by activating AMPK [22] ^,^ and the administration of H19 RNA increases the basal metabolic rate and protects against high-fat diet (HFD)- or leptin deficiency-induced obesity [23]. A recent study showed that the mouse lncRNA *Pair* and human *HULC*, which are associated with phenylalanine hydroxylase (PAH), are involved in the development of the inherited metabolic disorder phenylketonuria (PKU) [32]. *Pair*-knockout mice faithfully model human PKU, and targeting *HULC* significantly reduces PAH enzymatic activity in human induced pluripotent stem cell-differentiated hepatocytes [32]. Collectively, these findings suggest that lncRNAs may represent potential pharmaceutical targets for preventing and/or treating metabolic diseases, such as obesity, as well as inherited metabolic disorders.

Finally, our findings suggest an intriguing avenue through which researchers may identify signals or molecules that mediate the muscle-fat crosstalk for the lean phenotype observed in *Linc-RAM*-knockout mice. Previous studies suggested that endogenous metabolites regulate whole-body metabolic homeostasis by mediating interorgan crosstalk. For example, one study showed that an insufficient alanine supply mediates muscle-liver-fat signaling by upregulating FGF21 expression in the liver [33]. Further identification of such mediator(s) would greatly improve our understanding of the molecular mechanism underlying interorgan crosstalk for the maintenance of whole-body metabolic homeostasis, providing potential targets for the development of therapeutic drugs that can be used to prevent and/or treat metabolic diseases.
